# Impact of fresh grape juice on the pharmacokinetics of omeprazole: results of a food–drug interaction study

**DOI:** 10.3389/fphar.2024.1467805

**Published:** 2024-12-04

**Authors:** Tayyaba Iftikhar, Zafar Iqbal, Yasar Shah, Nauman Rahim Khan, Muhammad Abbas, Omer Shehzad, Fazal Hadi, Haseeba Sardar, Ali Abusharha, Maria Daglia, Haroon Khan

**Affiliations:** ^1^ Department of Pharmacy, Abdul Wali Khan University Mardan, Mardan, Pakistan; ^2^ Department of Pharmacy, University of Peshawar, Peshawar, Pakistan; ^3^ Department of Pharmacy, University of Science and Technology Kohat, Kohat, Pakistan; ^4^ Optometry Department, Applied Medical Sciences Collage, King Saud University, Riyadh, Saudi Arabia; ^5^ Department of Pharmacy, University of Naples Federico II, Naples, Italy; ^6^ International Research Center for Food Nutrition and Safety, Jiangsu University, Zhenjiang, China; ^7^ Department of Pharmacy, Korea University, Sejong, Republic of Korea

**Keywords:** food–drug interactions, omeprazole, grape juice, pharmacokinetics, therapeutic outcome

## Abstract

Grapes have been widely used for dietary ailments due to their attributed pharmacological activities. Resveratrol, the chief constituent of grapes, is responsible for their pharmacological benefits. However, apart from their beneficial effects, grapes have also recently been considered in drug interaction studies. This study investigated the pharmacokinetic profile of omeprazole administered alone compared to omeprazole administered with grape juice, with a prior intake of grape juice, for 1 continuous week. The study was conducted on two groups of healthy male volunteers [n = 12]. One group was orally administered 40 mg of omeprazole alone, while the other group was administered omeprazole with grape juice. Blood samples were analyzed for omeprazole concentration by a reverse-phase HPLC method. Co-administration of 40 mg omeprazole with grape juice significantly decreased the AUC_0-t_ and C_max_ by 32% and 34%, respectively, suggesting a role being played by grapes in the activation of P-glycoprotein and omeprazole metabolizing enzymes, including CYP3A4 and CYP2C19. In conclusion, the addition of grapes as a dietary supplement in patients taking omeprazole for the management of peptic ulcer symptoms may lead to a higher required dose of omeprazole.

## 1 Introduction

Medications for treating both major and minor ailments are now regarded as essential for human health. Many can be used with or without a prescription. Rational polypharmacotherapy includes the use of supportive medications not only to enhance recovery but also to treat one pathology in several ways or to prevent complications accompanying monotherapy (Miller et al., 2012). In some disease conditions, dietary choices can directly impact medication efficacy. For instance, managing diabetes may require balancing carbohydrate intake to ensure insulin works effectively (Reynolds and Mitri, 2024). It is always advisable to use fruits appropriately. However, the concurrent or extended use of certain medications alongside specific foods can disrupt the treatment process or cause unexpected toxicity or side effects (Reynolds and Mitri, 2024). This is primarily due to interactions between the drugs themselves or between the drugs and the food, potentially resulting in drug–drug or food–drug interactions (Alomar, 2014). Drug interactions can cause an alteration in the pharmacokinetic or pharmacodynamic effects of one drug through the co-administration of another drug, and food–drug interactions can cause an alteration in the disposition of the drug by interaction with dietary constituents (Rodríguez-Fragoso and Reyes-Esparza, 2013).

Interaction of natural products with drugs is a common problem faced during clinical practice. This kind of interaction is based on the same principles as those of drug–drug interactions. Researchers have identified several fruits and natural products that contain relevant compounds that alter the pharmacokinetics of co-administered drugs (Islam, 2014; Jáuregui-Garrido and Jáuregui Lobera, 2012; Ismail, 2009; Segal et al., 2014; Segal and Pilote, 2006). Biochemical mechanisms involve the modification of drug-metabolizing enzymes or transporters (Wolinsky and Williams, 2002). Enzymes such as the cytochrome P450 family play a pivotal role in the metabolism of drugs (Coleman, 2010; Rao et al., 2015). Interactions that inhibit or induce these enzymes can lead to unpredictable changes in drug concentrations and efficacy; for example, clarithromycin and erythromycin inhibited the metabolism of midazolam by inhibiting CYP3A4 (Qureshi, 2010). Additionally, transporters responsible for drug uptake and efflux across cell membranes may be affected by dietary components, further complicating the pharmacokinetics of drugs (Wang et al., 2007).

The extent of these interactions is frequently influenced by genetic factors, lifestyle choices, and diet. Factors such as smoking, alcohol consumption, and the intake of specific foods or supplements can exacerbate or mitigate drug interactions (Bushra et al., 2011; Wilkinson, 2005; Ayo et al., 2005). These potential impacts range from decreased pharmacological effectiveness to severe reactions and therapeutic failures.

Among these interactions, the interaction between grapes and omeprazole, a commonly used proton pump inhibitor, is an intriguing case study. Grapes are a common food source with a diverse nutritional profile. They are a rich source of polyphenols (resveratrol) in their natural state and as a juice. These are a group of substances that are structurally diverse and are present in various amounts. They are involved in the browning color of the grapes and wines and play a major role in the maturation of the wines. The phenolic compounds are found in the skin and seeds of grapes. The main compounds of this group are anthocyanins, flavanols (quercitin, myricetin, kaempferol), and stilbenes (resveratrol and a dimer of resveratrol, ε-viniferin), which have a significant effect on low-density lipoprotein (LDL) oxidation, oxidative stress furanocoumarins (bergamottin), and tannins (Barona et al., 2012; Tarko et al., 2008; Gambhire et al., 2011). Grape seed extract primarily contains phenolic compounds with notable pharmacological properties, including anti-inflammatory, anticancer, and antioxidant activities. Key compounds include catechin, epicatechin, flavonols, and others. In addition, as cited by Rockenbach et al. (2012), grape extracts consist of anthocyanins from the skin and procyanidins from the seeds (Barriga-Sánchez et al., 2022; Rockenbach et al., 2012).

Despite having some beneficial uses for health, some constituents of grapes have the potential to change the pharmacokinetics of certain drugs, such as omeprazole, by inhibiting drug-metabolizing enzymes and altering gastric pH (Kim et al., 2006). Therefore, the concurrent use of drugs that affect the substrates of such enzymes should be carefully monitored. Omeprazole belongs to the benzimidazole drug class. It has been comprehensively studied and is used extensively in the management of various acid-related gastric disorders (Varannes et al., 1994). It inhibits H^+^/K^+^ ATPase or proton pump in parietal cells by forming covalent bonds with cysteine residues through disulfide bridges on the alpha subunit of the H^+^/K^+^ ATPase pump, effectively inhibiting gastric acid secretion for up to 36 h. It is widely used in Zollinger–Ellison syndrome, peptic ulcer, and reflux esophagitis (Kromer et al., 1999). The bioavailability of omeprazole is increased by up to 60% with repeated doses, and it is one of the safest drugs with few adverse effects.

Omeprazole is metabolized by two subtypes of CYP enzymes, CYP2C19 and CYP3A4. The main contribution is from CYP2C19, but the role of CYP3A4 is not to be underestimated (Abid et al., 1994; Ogilvie et al., 2011). Interpopulation variation of omeprazole pharmacokinetics has been reported, and the main reason involved is phenotype variation of CYP2C19 isoforms (Baudhuin, 2012). Studies have also shown that omeprazole is a substrate of P-gp drug transporters, and, therefore, MDR1 polymorphism may also be a cause of pharmacokinetic variations of omeprazole (Marchetti et al., 2007). Omeprazole is generally well tolerated. The most common side effects, reported in a few cases, are headache, abdominal pain, nausea, diarrhea, vomiting, flatulence, nervousness, abnormal heartbeat, muscle pain, weakness, leg cramps, and water retention (McTavish et al., 1991; Cederberg et al., 1989; Andersson, 1991). Various studies have also reported interactions of different foods with omeprazole, such as the concomitant administration of grapefruit juice, which suppressed the AUC and C_max_ of omeprazole metabolite (Tassaneeyakul et al., 2000). Similarly, regular consumption of cranberry juice has been reported to alter the efficacy of omeprazole in *H. pylori* patients (Saltzman et al., 1994).

Given the widespread use of omeprazole for acid-related gastric disorders and the popularity of grapes as a dietary item, exploring their potential interactions is imperative. This research aims to shed light on the impact of grapes on the pharmacokinetics of omeprazole, contributing to our understanding of food–drug interactions and ultimately ensuring the safe and effective use of omeprazole in clinical practice when co-administered with grape juice.

## 2 Materials and methods

### 2.1 Chemicals

Omeprazole Reference Standard was a kind gift from Ferozsons Laboratories Limited, Nowshera. Internal standard pantoprazole was a kind gift from Medicraft Pharmaceuticals (Pvt) Ltd. Peshawar. HPLC-grade methanol was obtained from Sigma-Aldrich Dorset, United Kingdom. Distilled water was produced using a Millipore (Milford, United States) distillation apparatus. Omeprazole capsules (by Getz Pharma Pakistan, Batch no. 233c20) and analytical-grade potassium dihydrogen phosphate were purchased from Fischer Scientific (Leicester, United Kingdom). Sodium hydroxide was obtained from Merck (Darmstadt, Germany).

### 2.2 Grape source and type

Black seedless grapes (*Vitis vinifera*) imported from Iran were purchased from a local market in Peshawar, Pakistan. The grape juice was freshly prepared by blending at 35,000 RPM in a suitable blender (MOULINEX INFINYMIX LM91H) for 15 min, without adding sugar, water, or preservatives. The juice was used with pulp.

### 2.3 Instrumentation

The HPLC instrument used for analysis was the Perkin-Elmer series 200 (Norwalk, United States Of America). The HPLC comprised a pump (series 200), online degasser (series 200), manual injector (Rheodyne 7725i), Peltier column oven (Series 200), and UV-VIS detector (Series 200). The data obtained were analyzed by a Perkin–Elmer Totalchrom chromatography workstation (version 6.3.1) interfaced to the HPLC system through a network chromatography interface (NCI) 900. The column used was an Athena C18-WP, 5 µm, 100 A, Size: 4.6 × 150 mm, Serial no: LG102B01 (Dusseldorf, Germany), and a C18 (30 mm × 4.6 mm, 10 μm; cartridge (Norwalk, United States) was used as a guard column. The analytical balance used was an electronic balance from Nova Biochem (Merck) TP-214. The Touch Vortexmixer was obtained from Fisher Scientific Model: 232, Germany. A high-quality centrifuge (-K280, Centurion Scientific, United Kingdom) was used to separate plasma from the whole blood with a speed limit from 1,000 rpm to 10,000 rpm and temperatures from 15°C to −9°C. The pH meter was a PHS-25CW microprocessor from Banteinstrument. Double-distilled water used to prepare various aqueous solutions was freshly prepared through a Millipore RO water distillation system (United States). The HPLC mobile phase and other solutions were filtered through 0.45-µm polyvinylidene fluoride (PVF) membrane filters. Stock solutions (1 mg/mL) were prepared in methanol and further diluted using the mobile phase.

### 2.4 Extraction

Methanol was chosen as an extraction solvent, as described previously (Ahmad et al., 2011). Methanol (750 µL) was added to plasma (250 µL) and centrifuged at 5,000 rpm for 5 min at 0°C. The clear supernatant was transferred to an Eppendorf tube and subjected to nitrogen evaporation. The residue was dissolved in 250 µL methanol and vortexed for 1 min, and 20 µL of the extracted plasma was injected into the HPLC.

### 2.5 Subject population

#### 2.5.1 Inclusion criteria

Normal healthy male volunteers (n = 12) with no history of illness were selected and divided into two equal groups (n = 6 in each group, aged 20–25 years). More than 20 volunteers were screened using various physical and biochemical tests such as lipid profiles, liver function tests (LFTs), renal function tests (RFTs), blood pressure (BP), and blood glucose levels. Electrocardiography (ECG) was also performed for all subjects, and those with perfectly normal results were included in the study.

#### 2.5.2 Exclusion criteria

Subjects having any disease such as coronary heart disease, renal disease, diabetes mellitus, hepatic impairment, or any other systemic pathology were excluded and not considered for the study. Subjects who were obese and smokers were also excluded. Older subjects at risk of certain diseases, including coronary heart disease or chronic renal failure, and those with special diets were also not considered for the study.

### 2.6 Study design and regimen

The open-label and randomized study design was used to conduct the study in normal, healthy male volunteers. All volunteers were directed not to take any type of medication, including herbal medicines, for 2 weeks prior to clinical trials. Volunteers were divided into two groups. In treatment period-I, after overnight fasting, volunteers in group A received omeprazole (40 mg) alone, and volunteers in group B, to whom grape juice had been given for 3 consecutive days, were administered omeprazole (40 mg) along with grape juice (≈ 250 mL) on the fourth day. After a 1-week washout period, in treatment period-II, the volunteers in group A, who had received grape juice for 1 week prior, were administered omeprazole (40 mg) along with grape juice (≈ 250 mL) on the fourth day. The volunteers in group B were given omeprazole (40 mg) alone. A standard breakfast comprised fried eggs, tea, and bread, and lunch comprised bread, chicken curry, and salad, provided 2 h and 6 h following administration of the drug, respectively.

### 2.7 Pharmacokinetics assessment

Blood samples (≈ 3 mL) were collected from each volunteer before the administration of the drug, as a blank, and following the administration of the drug at 0 h, 0.5 h, 1 h, 1.5 h, 2 h, 3 h, 4 h, 6 h, 8 h, and 24 h, in EDTA blood collection tubes. The samples were centrifuged at 1,000 rpm for 5 min, and plasma was separated and kept in a freezer at −20°C until analysis. Each sample was prepared by adding 200 µL plasma, 50 µL internal standard (pantoprazole), 150 µL distilled water, and 600 µL methanol and then vortexed for 1 min. It was then centrifuged at 5,000 rpm for 5 min at −3°C. The supernatant was removed and evaporated in a nitrogen flux and then reconstituted in the mobile phase up to 0.5 mL and stored in a refrigerator.

The samples were analyzed using a validated HPLC/UV-Vis chromatographic method developed for plasma sample analysis. The plasma samples were thawed on ice at room temperature and processed for analysis using the developed method. A calibration curve was plotted for varying concentrations of omeprazole, keeping the concentration of pantoprazole constant, and used as an internal standard. The concentration range for omeprazole was 0.1 μg/mL, 0.2 μg/mL, 0.4 μg/mL, 0.6 μg/mL, 0.8 μg/mL, 1.0 μg/mL, 1.2 μg/mL, and 1.5 μg/mL. The concentration of pantoprazole was kept at 1 μg/ml as an internal standard. The concentration of omeprazole was 1 μg/mL, 1.5 μg/mL, and 2 μg/mL, and the concentration of internal standard was 1 μg/mL.

### 2.8 Data analysis

The pharmacokinetic parameters of plasma omeprazole were calculated for each individual, using non-compartmental analysis of plasma concentration–time data with the help of PK-Summit^®^ software. C_max_, the maximum plasma concentration, and t_max_, the time to reach the maximum plasma concentration, were directly calculated from the analyzed data. The terminal half-life was calculated using the equation 0.693/kel, where kel represents the value of the elimination rate constant. The area under the plasma concentration–time curve was calculated according to the trapezoidal rule over the time interval 0 to the last measurable concentration (AUC_0–t_) and subsequent extrapolation to time infinity (AUC_0–∞_). The first moment of the plasma concentration vs. time curve (AUMC) is the plasma concentration multiplied by time vs. the time curve calculated by the same trapezoidal rule. The total body clearance (CL/F) was calculated as dose/AUC_0–∞_. The volume of distribution was calculated by multiplying total body clearance with the mean residence time (MRT). Statistical analysis was performed by paired t-test, and the pharmacokinetic parameters of omeprazole with water were compared with the parameters obtained from omeprazole administered with grape juice. A *p*-value of < 0.05 was considered significant.

## 3 Ethical approval

The clinical trials using human healthy volunteers were conducted in accordance with the “Helsinki Declaration.” Before conducting the study, approval from the local ethical committee of the Department of Pharmacy, University of Peshawar, KPK, Pakistan, Ref. No. “EA/Pharm/UOP/1131” was obtained, and written informed consent was obtained from all participants enrolled.

## 4 Results

### 4.1 Subject information

Healthy male Pakistani volunteers (n = 12) were selected based on normal results of the biochemical screening tests. These tests included hemoglobin (Hb), hepatitis B and C, bilirubin, SGPT, hyperlipidemia-related tests such as low-density lipoprotein (LDL), high-density lipoprotein (HDL), and triglycerides. Blood pressure and heart rate values were also measured, and the individuals were found to be normal and healthy according to the selection criteria. The mean height, weight, and BMI values were also calculated, and all of the volunteers were found to be normal according to the standard of the Pakistani people, as shown in Table 1. The small SD of age, height, and weight indicate that all of the volunteers were of a similar physique, and, thus, the PK behavior may not show high variability due to the physiological parameters.

**TABLE 1:** Demographic data of the volunteers.

Volunteers	Age (years)	Height (cm)	Weight (lbs)	BMI (lbs/in^2^) × 703
1	25	151	136.7	21.9
2	30	179	149.9	24.5
3	28	173	138.9	23.9
4	27	177	147.7	24.6
5	29	181	148.5	24.5
6	24	182	158.4	22.3
7	26	178	141.5	23.9
8	23	168	156.8	23.1
9	22	177	147.1	22.4
10	29	180	131.8	21.8
11	22	163	157.8	22.9
12	25	170	154	24.0
Mean	25.8	173.2	147.4	23.3
St. Dev.	±2.79	±9.06	±8.7	±1.04
Range	22–30	162–183	132–158.4	21.94–24.65

### 4.2 Pharmacokinetic analysis

**FIGURE 1:**
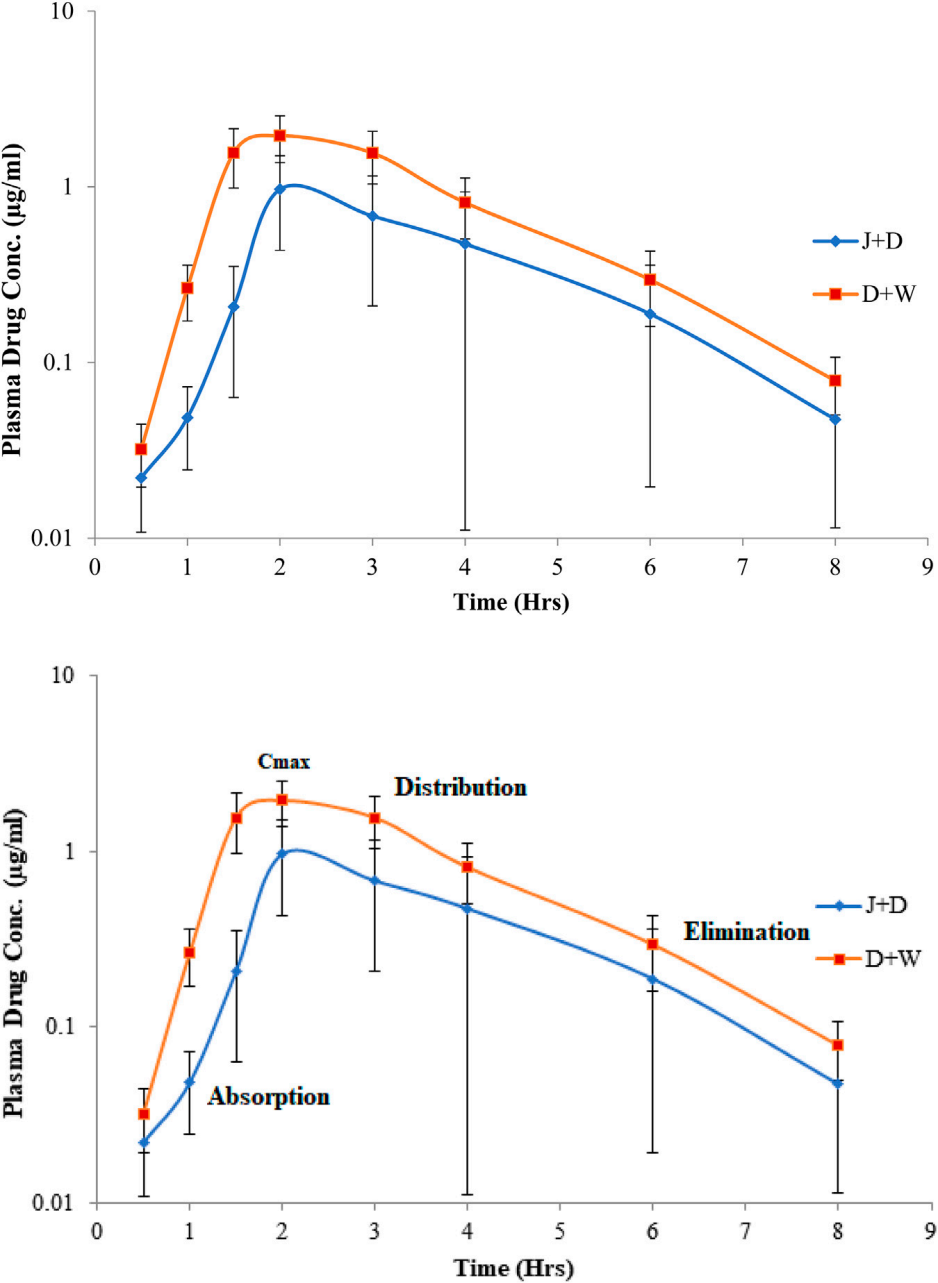
Logarithmic plasma drug concentrations vs. time plot of omeprazole administered alone (D (drug) + W (water)) and co-administered with grape juice (J (juice) + D (drug)).

Plasma samples were analyzed using HPLC-UV methods. The values of the pharmacokinetic parameters of omeprazole were found to match those from previously reported research. Omeprazole plasma drug concentrations were plotted as a function of time, as shown in Figure 1, for omeprazole analyzed with co-administered grape juice and water. The plots show that omeprazole and its metabolites follow a one-compartment model. The maximum plasma omeprazole concentration (C_max_) and the time to C_max_ (t_max_) were calculated directly from the curve. All pharmacokinetic parameters including AUMC, AUC_0-t_ and AUC_0-∞_, Cl, Vd, MRT, t_1/2_, K_12_, K_21_, and K_10_ were calculated. The pharmacokinetic parameters of omeprazole administered with water as a control, calculated using non-compartmental models, are summarized in Table 2, and the parameters of omeprazole administered with fresh grape juice are summarized in Table 3. The comparative pharmacokinetics of omeprazole administered alone and with grape juice are given in Table 4.

**TABLE 2:** Pharmacokinetic parameters of omeprazole administered with water (n = 6).

PK parameters		Omeprazole alone	
		Mean	± SD
1	C_max_ (µg/mL)	1.953	0.581
2	T_max_ (hr)	2	0.00
3	${\left[\text{AUC}\right]}_{0}^{t}$ (µg.hr/mL)	5.84	1.56
4	${\left[\text{AUC}\right]}_{0}^{\infty }$ (µg.hr/mL)	6	1.54
5	${\left[\text{AUMC}\right]}_{0}^{\infty }$ (µg.hr*hr/mL)	19.6	4.91

**TABLE 3:** Pharmacokinetic parameters of omeprazole administered with fresh grape juice (n = 6).

PK parameters		Omeprazole with juice	
		Mean	± SD
1	C_max_ (µg/mL)	1.018	0.535
2	T_max_ (h)	2.09	0.30
3	${\left[\text{AUC}\right]}_{0\;}^{t}$ (µg.h/mL)	2.83	1.88
4	${\left[\text{AUC}\right]}_{0}^{\infty }$ (µg.h/mL)	2.91	1.92
5	${\left[\text{AUMC}\right]}_{0}^{\infty }$ (µg.h*h/mL)	10.36	7.23

**TABLE 4:** Comparative pharmacokinetics of omeprazole administered alone and with grape juice.

Sr. No	PK parameters	Omeprazole with juice	Omeprazole	*p*-value	Ratio (OMP + juice/OMP)
1	C_max_ (µg/mL)	1.018	1.953	0.001	0.52124936
2	T_max_ (h)	2.09	2	0.9569378	1.045
3	${\left[\text{AUC}\right]}_{0\;}^{t}$ (µg.h/mL)	2.83	5.84	0.000	0.48458904
4	${\left[\text{AUC}\right]}_{0}^{\infty }$ (µg.h/mL)	2.91	6	0.007	0.48500000
5	${\left[\text{AUMC}\right]}_{0}^{\infty }$ (µg.hr*h/mL)	10.36	19.6	0.001	0.52857143

AUC, area under the curve; AUMC, area under the first-moment curve; MRT, mean residence time; Vd, volume of distribution; E, elimination; D, distribution; A, absorption; Cl, clearance.

#### 4.2.1 Peak plasma concentration (C_max_)

**FIGURE 2:**
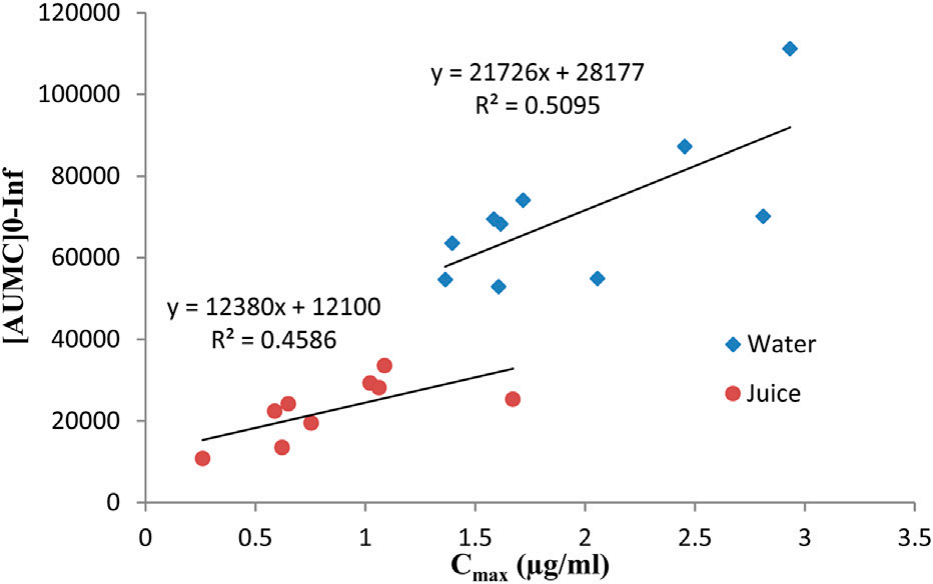
Correlation between the [AUMC] 0–Inf and C_max_ when omeprazole was administered alone with water or with grape juice.

The mean plasma concentration of omeprazole after administration of 40 mg omeprazole with water was 1.953 ± 0.581 μg/mL. In contrast, the mean plasma concentration of 40 mg omeprazole administered with 250 mL fresh grape juice was 0.847 ± 0.385 μg/mL. The peak plasma concentration of omeprazole administered with grape juice compared to omeprazole administered with water was significantly decreased (*P* > 0.001). The *p*-value obtained from the paired t-test was 0.001. A positive correlation was observed between C_max_ and AUMC of omeprazole administered with water and juice, and the values found were 0.50 and 0.45, respectively, as shown in Figure 2.

#### 4.2.2 Time to reach C_max_ (t_max_)

**FIGURE 3:**
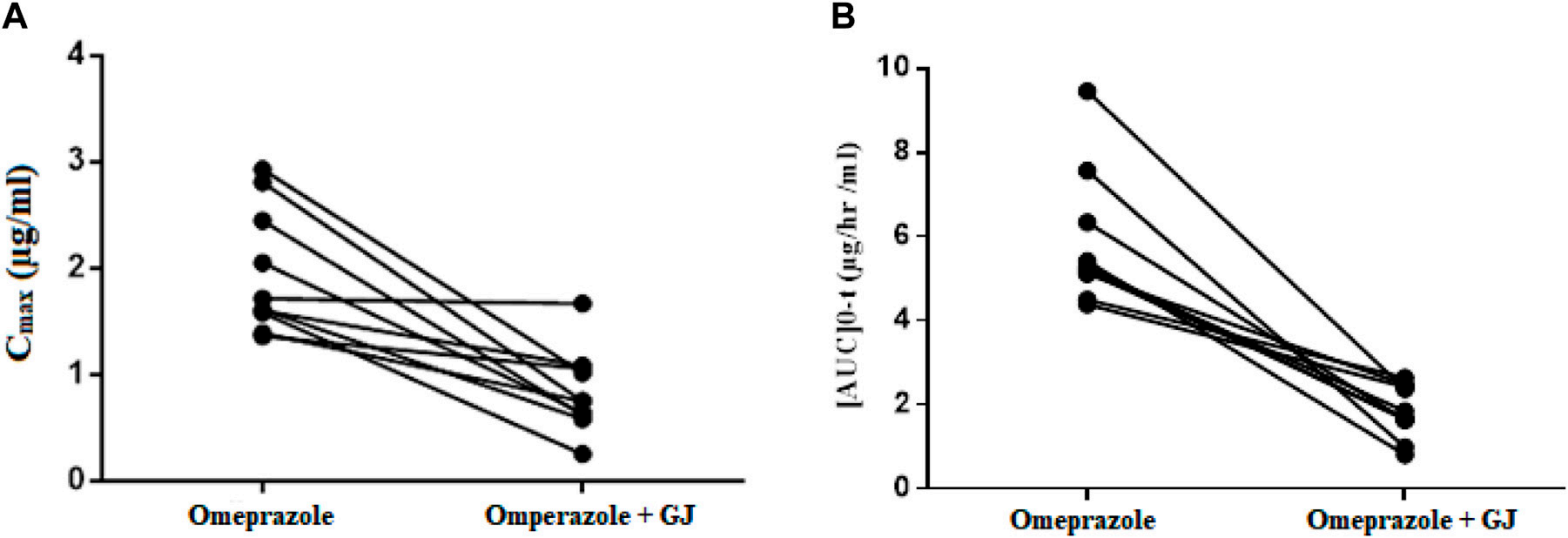
**(A)** C_max_ of OMP (40 mg) **(B)** [AUC] 0-t of OMP (40 mg), administered alone or with grape juice.

The mean time to reach the maximum plasma concentration of 40 mg omeprazole administered with water was 120.00 ± 0.00 min. The mean time to reach the maximum plasma concentration of 40 mg omeprazole co-administered with fresh grape juice was 125.45 ± 18.09 min (Figure 3A).

#### 4.2.3 Area under the plasma concentration–time curve

**FIGURE 4:**
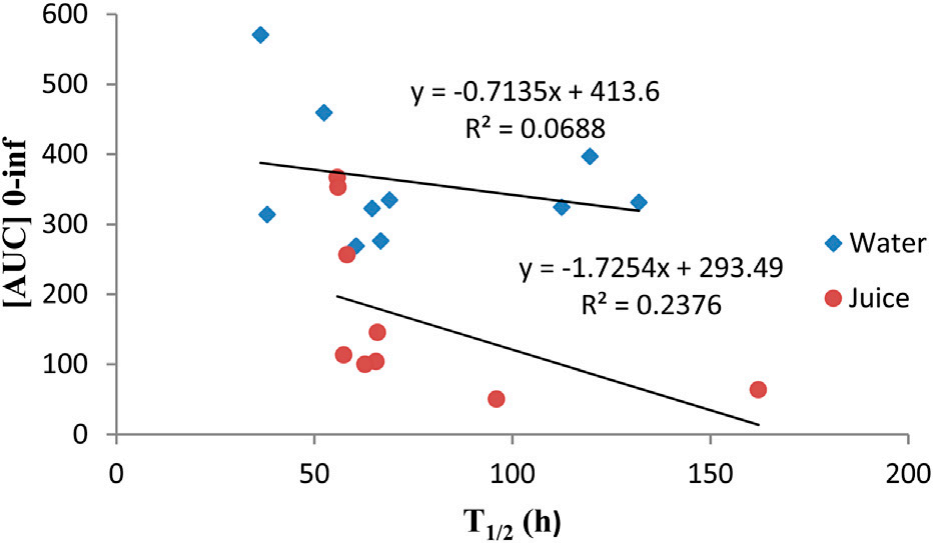
Correlation between the [AUC] 0–Inf and t_1/2_ when omeprazole was administered alone with water or with grape juice.

The value of area under the plasma concentration curve for omeprazole with water was calculated according to the trapezoidal rule, from time 0 to the last measurable plasma concentration, and was subsequently extrapolated for the time at infinity. The values for ${\left[\text{AUC}\right]}_{0\;}^{t}$ and ${\left[\text{AUC}\right]}_{0\;}^{\infty }$ for 40 mg omeprazole administered with water were 5.84 ± 1.56 μg .h/mL and 6 ± 1.54 μg .h*h/mL, respectively. The values for the same parameters for 40 mg omeprazole administered with fresh grape juice were found to be 2.83 ± 1.88 μg .h/mL and 2.91 ± 1.92 μg .h/mL, respectively, as shown in Figure 3B. A significant decrease (*P* < 0.000) and (*P* < 0.007) in area under the plasma concentration–time curve for ${\left[\text{AUC}\right]}_{0\;}^{t}$ and ${\left[\text{AUC}\right]}_{0\;}^{\infty }$ , respectively, was observed with omeprazole administered with grape juice. AUC was found to be positively correlated with the t_1/2_ of omeprazole administered with water and juice, indicating a decrease in AUC with a subsequent decrease in t_1/2_, as shown in Figure 4.

## 5 Discussion

In the present research, the pharmacokinetic parameters of omeprazole were found to be in accordance with previously reported parameters (Rost and Roots, 1996; Yin et al., 2004; Andersson et al., 2000; Hu et al., 2007). However, a wide range of C_max_ has been observed in different populations, resulting in inter-individual variability (Andersson, 1991; Ramsjö et al., 2010). The t_max_ of omeprazole was analyzed as 2 h, and the range of t_max_ already reported is 2–3 h. The results were found to be in accordance with values obtained from the Chinese population (Motevalian et al., 1999). The values of AUC were found to be higher than average AUC values reported in different populations, and the reason could be due to low levels of omeprazole metabolites formed.

With the developing awareness of interactions between drugs and dietary constituents, much research is being conducted on the effects of concomitant administration of drugs and food on drug bioavailability. To date, not much work has been done on the effects of grape juice on drugs, although it contains resveratrol, which has been found to have various pharmacological benefits and is being extensively used worldwide. Owing to its ability to alter the CYP450 (CYP3A4) and P-gp, grape juice has drawn the attention of researchers exploring interaction studies (Alfaras et al., 2010; Brand et al., 2006; Moon et al., 2006). Hence, the dietary constituents modulating P-gp and metabolic enzymes have been under wider consideration in the past few years (Scripture and Figg, 2006). These results show that a constant intake of grape juice for 4 days reduced the bioavailability of omeprazole, which may lead to therapeutic failure or dose adjustment, as the pharmacokinetics of omeprazole after co-administration with grape juice taken once a day continuously for 1 week were observed.

The normal pharmacokinetic values of drugs metabolized by CYP3A4 may vary, and the reason underlying this variability may be inter-individual variability in CYP3A iso-enzymes (Pollock, 1996). The measured pharmacokinetic parameters of omeprazole were found to be more or less similar to previously calculated values (Rost and Roots, 1996; Andersson et al., 2000). Some of the pharmacokinetic parameters of the omeprazole administered with water and grape juice were found to be significantly different.

The value of C_max_ and AUC were found to be markedly decreased when the omeprazole was administered with grape juice. The decrease in the C_max_ of omeprazole may be due to the induction of CYP2C19 or CYP3A4, which might have resulted in a rapid metabolism of omeprazole before reaching t_peak_ plasma concentration. Further work may be conducted to assess the concentration of metabolites of omeprazole after co-administration of omeprazole and grape juice. Very similar results were shown when cyclosporin, which is also a substrate of CYP3A4 and P-gp, was administered with purple grapes; the C_max_ and AUC were reduced up to 30% (Oliveira-Freitas et al., 2010). The author attributed the decreased bioavailability of cyclosporin to the enhanced activity of CYP3A4 and P-gp. Because P-gp provides an important contribution to the poor absorption of many drugs, our results support the conclusion of the previous work, stating that grape juice is an inducer of P-gp in the small intestine, leading to decreased drug absorption (Oliveira-Freitas et al., 2010). Another study analyzed the concomitant administration of grape juice with midazolam, and the bioavailability of midazolam was increased due to the induction of CYP3A4 (Nishikawa et al., 2004). The main metabolizing enzyme involved in the pharmacokinetics of omeprazole is CYP2C19 (Ota et al., 2014). The effect of grape juice on CYP1A2 enzymes has been reported in previous research in which the reduced AUC of phenacetin (a substrate of CYP1A2 enzymes) was analyzed following the administration of grape juice in a group of healthy Chinese subjects (Dong et al., 1999).

As the values of AUC (0-t) and AUC (0-∞) for omeprazole administered with grape juice were also found to decrease significantly (0.000 and 0.007, respectively), the relative decrease in the bioavailability of omeprazole may be credited to the induction by grape juice of P-gp and omeprazole metabolizing enzymes in the small intestine. T_max_ values were slightly increased after co-administration with juice, exhibiting a slight increase in drug entity exposure within the body.

The half-life of omeprazole was not affected by a regular intake of 250 mL of grape juice for 1 week before administration of omeprazole, which indicates that the enzyme induction may occur primarily in the intestinal wall. The mean value of clearance was increased, which may be due to the decrease in the AUC of omeprazole and the increase in metabolism, resulting in low bioavailability.

Therefore, the dose of omeprazole should be adjusted when taken concurrently with grape juice to avoid extended treatment durations and poor outcomes. Conditions such as Zollinger–Ellison syndrome and peptic ulcer disease require sufficient bioavailability of omeprazole; inadequate levels can reduce its efficacy (Ito et al., 2023). Peptic ulcer disease (PUD) is frequently associated with *Helicobacter pylori* bacterial infection, which can be aggravated by NSAIDs. Addressing *H. pylori* infection often requires including omeprazole or other proton-pump inhibitors in the regimen (Albayrak et al., 2023). Because *H. pylori* grows at a neutral pH, elevating the gastric pH with proton-pump inhibitors like omeprazole helps suppress its growth (Dawood and Mamdooh, 2021).

The importance of omeprazole can be manifested from its role in various treatment regimens. For instance, in the management of ankylosing spondylitis, omeprazole is used alongside diclofenac to mitigate gastrointestinal issues associated with NSAIDs (Bannwarth and Zerbib, 2006). During the COVID-19 pandemic, patients receiving dexamethasone for inflammation were also prescribed omeprazole to prevent gastric complications caused by the steroids (Singh and Kothari, 2022). This underscores the crucial role omeprazole plays in protecting against drug-induced gastric side effects across different treatment scenarios.

In summary, conditions characterized by pathologic hypersecretion, such as Zollinger–Ellison syndrome, peptic ulcer disease, primary hyperparathyroidism, and multiple endocrine neoplasia syndromes, involve excessive production of gastric acid that can result in adverse clinical outcomes (Barreras and Donaldson, 1967; Norton et al., 2008). Omeprazole plays a crucial role in managing these conditions by lowering gastric acid secretion, alleviating symptoms, facilitating healing, and preventing complications. It is important to note that to achieve optimal plasma levels of omeprazole, either the intake of grape juice must be carefully managed, or the dosage of omeprazole must be adjusted accordingly.

## 6 Conclusion

In conclusion, consistent grape juice consumption modifies the pharmacokinetics of omeprazole and dramatically reduces its bioavailability. These results can be further assessed by performing *in-vitro* examinations of enzyme induction or inhibition investigations. Given the current research and findings, it is advised that people using omeprazole for peptic ulcers should avoid drinking grape juice on a daily basis or that the dosage should be changed to maintain efficacy.

## Data Availability

The raw data supporting the conclusions of this article will be made available by the authors, without undue reservation.
